# Biotin-cGMP and -cAMP are able to permeate through the gap junctions of some amacrine cells in the mouse retina despite their large size

**DOI:** 10.3389/fopht.2023.1334602

**Published:** 2024-01-15

**Authors:** Chunxu Yuan, Luca Gerhards, Ilia A. Solov’yov, Karin Dedek

**Affiliations:** ^1^ Animal Navigation, Institute for Biology and Environmental Sciences, Carl von Ossietzky Universität Oldenburg, Oldenburg, Germany; ^2^ Institute of Physics, Carl von Ossietzky Universität Oldenburg, Oldenburg, Germany; ^3^ Research Center Neurosensory Science, University of Oldenburg, Oldenburg, Germany; ^4^ CeNaD – Center for Nanoscale Dynamics, University of Oldenburg, Oldenburg, Germany

**Keywords:** retina, gap junction, electrical synapse, horizontal cell, amacrine cell, mouse

## Abstract

Gap junctions transmit electrical signals in neurons and serve metabolic coupling and chemical communication. Gap junctions are made of intercellular channels with large pores, allowing ions and small molecules to permeate. In the mammalian retina, intercellular coupling fulfills many vital functions in visual signal processing but is also implicated in promoting cell death after insults, such as excitotoxicity or hypoxia. Conversely, some studies also suggested a role for retinal gap junctions in neuroprotection. Recently, gap junctions were also advocated as conduits for therapeutic drug delivery in neurodegenerative disorders. This requires the permeation of rather large molecules through retinal gap junctions. However, the permeability of retinal networks for molecules >0.6 kDa has not been tested systematically. Here, we used the cut-loading method and probed gap junctional networks in the mouse retina for their permeability to cGMP and cAMP coupled to Biotin, using the well-characterized tracer Neurobiotin as control. Biotin-cGMP and -cAMP have a molecular weight of >0.8 kDa. We show that they cannot pass the gap junctions of horizontal cells but can permeate through the gap junctions of specific amacrine cells in the inner retina. These amacrine cells do not comprise AII amacrine cells and nitric oxide-releasing amacrine cells but some unknown type. In summary, we show that some retinal gap junctions are large enough to let molecules >0.8 kDa pass, making the intercellular delivery of therapeutic agents – already successfully exploited, for example, in cancer – also feasible in neurodegenerative diseases.

## 1 Introduction

Gap junctions or electrical synapses are clusters of intercellular channels connecting the interior of two adjacent cells, allowing for the exchange of ions, second messengers, and other signaling molecules (<1 kDa) (1, 2). Gap junctions, formed by connexin proteins, are widespread in the central nervous system and particularly abundant in the mammalian retina, where 11 different connexin isoforms were reported to be expressed [reviewed in (3)]. All major classes of retinal neurons (photoreceptors, horizontal, bipolar, amacrine, and ganglion cells) and glia cells form coupled networks in the retina (4–6), which contribute to visual signal processing by playing an essential role in dim light vision (7–9), noise reduction (10, 11), spike synchronization (12, 13), and regulation of receptive field size (14, 15).

Gap junctions are essential for visual processing and are also shown to be involved in secondary cell death in the retina by potentially spreading toxic molecules from dying cells to coupled neighbors (16, 17). For example, induction of apoptotic cell death in individual retinal ganglion cells leads to the loss of neighboring ganglion and amacrine cells. In contrast, the blockade of gap junctions prevents this so-called “bystander effect” (16, 18). In addition to their spreading death signals, gap junctions were discussed as neuroprotectors (19), saving neighboring cells from insults. For example, inhibitors of gap junctions were shown to cause apoptosis (20); also, upregulation of connexin36 in retinal neurons was established to protect from secondary cell loss, while loss of connexin36 was reported to promote secondary cell death (21). These findings have identified gap junction proteins as promising therapeutic targets for neuroprotection (reviewed in 22).

Recently, gap junctions were also advocated as promising new routes for therapeutic drug delivery (23–26). For example, gap junction-containing liposomes were used in cells to successfully deliver chemotherapeutics to breast cancer cells (26), and gap junctions were exploited to send small interfering RNAs (siRNA) from one cell to another in cultured cells (27). However, knowledge on the potential use of gap junction-mediated drug delivery in neurodegenerative disorders is scarce, and a deepened understanding of the permeability of neuronal gap junctions is needed. Here, we used the mouse retina as a test system and evaluated the permeability of two intracellular messengers (cGMP, cAMP), which can exert various functions in retinal neurons, including activation of protein kinases, ion channels, and transcription factors (28). Both cGMP and cAMP were conjugated to Biotin, which is known to permeate through gap junctions and can easily be visualized by fluorophore-coupled streptavidin. We found that - despite their large size of >0.8 kDa - Biotin-conjugated cAMP and cGMP were able to pass some gap junctions in the inner retina, while not passing to gap junctions between horizontal cells and between AII amacrine cells. This study shows that some neuronal gap junctions can permeate surprisingly large substances, opening up an avenue for drug delivery in neurodegenerative disorders.

## 2 Methods

### 2.1 Animals and tissue preparation

Animals were maintained under a 12 h light/dark cycle with food and water *ad libitum*. All procedures were approved by the local animal welfare committee [*Niedersächsisches Landesamt für Verbraucherschutz und Lebensmittelsicherheit*, KDE TSG4 (3)] and were in accordance with the law on animal protection issued by the German Federal Government (*Tierschutzgesetz*). C57BL6/J mice (aged 4 to 6 months, both sexes) were dark-adapted for 1.5 h before euthanasia with CO_2_ to equilibrate their adapted state. After cervical dislocation, eyes were rapidly removed and transferred to hydrogen carbonate-buffered Ames’ solution (A1420-10X1L, Sigma-Aldrich, MO, USA) equilibrated with carbogen (95% O_2_/5% CO_2_) and maintained at 32°C. The cornea, lens, and vitreous body were carefully removed. The choroid fissure was identified on the sclera (29), and a reference cut was made on the ventral temporal side of the eye cup with curved scissors to keep track of the retinal orientation.

The eyecup (containing retina, pigment epithelium, choroid, and sclera) was preincubated in Ames’ solution for 20 mins with or without 50 μM meclofenamic acid (MFA, M4531, Sigma-Aldrich).

### 2.2 Cut-loading

After preincubation, the eye cup was briefly removed from the Ames’ solution and cut along the nasal, temporal, ventral, and dorsal side with a size 11 scalpel blade coated with 15.5 mM N-(2-aminoethyl) biotinamide hydrochloride (0.5% Neurobiotin™ Tracer, SP-1120, Vector Laboratories, CA, USA), 15.5 mM Biotin-conjugated cGMP (00021, Biotium) or 15.5 mM Biotin-conjugated cAMP (00020, Biotium). The eyecup was incubated with the tracer for 30 seconds and then immersed in Ames’ solution. The tracers were allowed to diffuse for 10 min. Then, the retina was quickly dissected from the eyecup, mounted (ganglion cells up) onto black filter paper (0.8 µm pore size, MF-Millipore™, Ireland), and fixed with 2% paraformaldehyde supplemented with 3% sucrose (w/v, diluted in 0.1 M phosphate buffer, PB) at room temperature for 30 min.

### 2.3 Immunostaining

Retinas were washed in 0.1 M PB before blocking in 10% donkey serum (diluted in 0.1 M PB with 0.5% Triton-X100) for one hour and then incubated with Alexa Fluor™ 568 Streptavidin (1:250; S11226, Invitrogen) overnight. In some experiments, tracer visualization was combined with immunohistochemistry. Whole-mounts were incubated in primary antibodies (Pax6, 1:25, mouse; Developmental Studies Hybridoma Bank, RRID: AB_528427; GABA, 1:250, rabbit; A2052, Sigma Aldrich, RRID: AB_477652; NOS1, 1:500, mouse, sc5302, Santa Cruz Biotechnology, RRID: AB_626757) for three days at 4°C. After extensive washing in 0.1 M PB, the retinas were incubated for two days at 4°C with the secondary antibodies (Donkey Anti-Mouse IgG H&L Alexa Fluor^®^ 488, ab150105, abcam, RRID: AB_2732856; Donkey anti-Rabbit IgG H&L Alexa Fluor™ 488, A-21206, Invitrogen, RRID: AB_2535792). To visualize cell nuclei, incubation in 4′,6-diamidino-2-phenylindole (DAPI, 1:1000, ab228549, Abcam) followed. After several washing steps, retinas were mounted on slides and coverslipped with an aqueous mounting medium (Vectashield, Vector Laboratories).

### 2.4 Microscopy

Images were collected at similar retinal eccentricities using a confocal laser scanning microscope (Leica TCS SP8) and a 20x objective (HC PL APO 20x/0.7 imm.). Settings were kept constant for one set of experiments; confocal stacks (1,024×1,024 pixels) were acquired from the outer plexiform layer to the ganglion cell layer (1 µm steps) at a zoom of 1.

### 2.5 Image analysis and quantification

Image analysis was done with Fiji (30). In brief, image stacks were background subtracted (rolling ball radius of 50 pixels) and contrast-enhanced using Fiji tools. Evenly spaced Neurobiotin+/Biotin+ cells with large somata in the distal inner nuclear layer were identified as horizontal cells which was confirmed by calbindin labeling (see **Supplementary Figure 1**). Pax6+/Neurobiotin+ cells in the proximal inner nuclear layer were identified as amacrine cells. For intensity plots, Pax6+ and Neurobiotin+ cells were identified by the *Colocalization Highlighter* tool (using Li as an automated threshold), and their mean pixel intensity and distance from the cutting edge were measured. For the Neurobiotin channel, intensities were normalized per cutting site (evaluated at 0.5 to 1.1 mm from the optic nerve head) and then plotted as relative intensity against distance from a cut in OriginPro 2021 (OriginLab). The intensity plot was fit with a single exponential function (Equation 1), deriving a length constant λ from the fit:


(1) $$y\;=\;A1*\exp\left(-\frac{x}{\lambda }\right)+\;y0$$

Only fits with R^2^ values >0.8 were considered. Differences between length constants were determined by a Mann Whitney U test.

Cells were counted in regions of interest of 113.84 µm × 113.81 µm to determine the number of labeled cells/mm^2^ with the *Cell Counter* plugin in Fiji. To test for significant differences in the number of labeled cells, one-way ANOVA was performed (GraphPad Prism 10.1, Dotmatics) with “substance” as a factor and *posthoc* corrections for multiple comparisons (Dunnett’s T3 multiple comparisons test). The alpha level was 0.05 for all statistical tests. Values are always given as mean ± standard deviation of the mean. If the p-value is below 0.0001, it is given as p < 0.0001; in all other cases, exact p-values are given.

### 2.6 Quantum chemistry optimization of molecular structures

Quantum chemistry computations were performed to determine the optimized structures of Biotin-cGMP, Biotin-cAMP, and Neurobiotin employing the ORCA 5.0.0 (31) software package. The calculations utilized the TPSSh GGA functional with the def2-SVP basis set (32, 33) and the resolution of identity approximation (RIJCOSX) with the def2/J auxiliary basis. The calculations were carried out with account for the D3 dispersion correction (34, 35). Since the dispersion interactions are not described accurately by the GGA functionals, Grimme’s correction was used (34).

To investigate possible conformers of Biotin-cAMP/-cGMP and Neurobiotin, we employed the software package CREST (36, 37) (Conformer-Rotamer Ensemble Sampling Tool). Metadynamics exploring the conformational space was performed at 350 K. Conformers were sorted according to their energy, and the structures within an energy window of 10.0 kcal/mol from the most stable conformer were considered. For Biotin-cAMP, 899 conformers were found, while for Biotin-CGMP 395 conformers could be determined; in the case of Neurobiotin 471 conformers were found. All the molecules revealed high flexibility, which is expected for the long aliphatic part of each molecule. The ten most stable conformers were selected, and the geometry of these conformers was further optimized using the TPSSh/def2-SVP/D3 method. The size measurements along the three principal directions were performed for the most stable conformer of each molecule.

Optimizations without dispersion correction were carried out to qualitatively investigate the structure of Biotin-cAMP/-cGMP and Neurobiotin geometries in solution. Less stacked configurations due to the lack of weak van-der-Waals self-interactions were found.

## 3 Results

### 3.1 Tracer coupling in the outer retina

**Figure 1:**
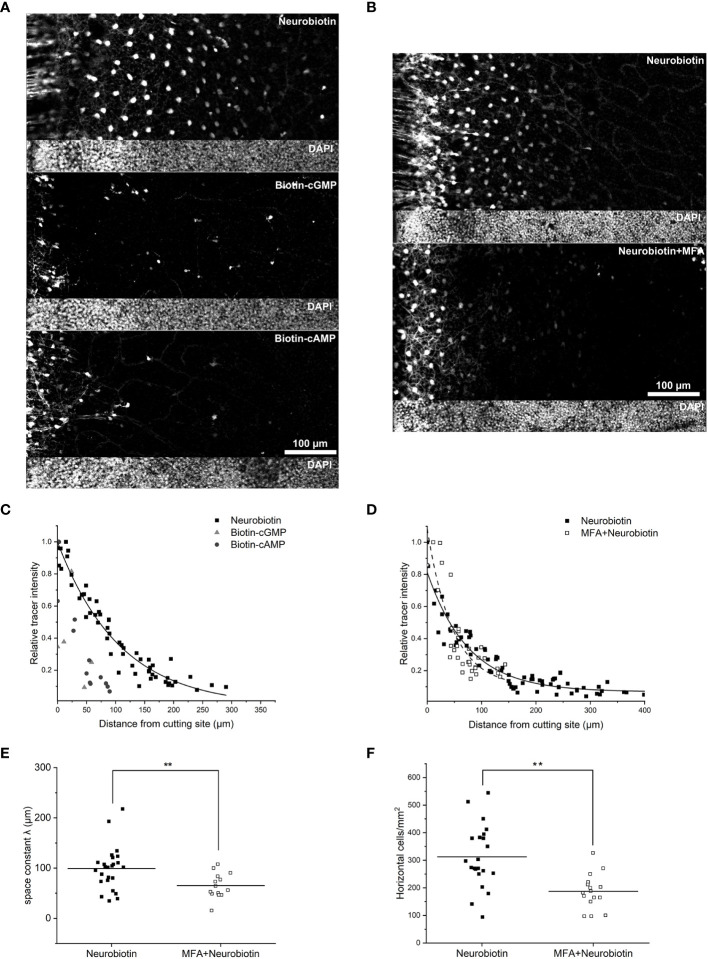
Gap junctions of mouse horizontal cells are permeable for Neurobiotin but not for Biotin-cGMP and -cAMP. **(A)** Diffusion of tracers, cutting site on the left. **(B)** MFA reduced the spread of Neurobiotin in the horizontal cell network. **(C)** The relative tracer intensity of Neurobiotin, Biotin-cGMP, and Biotin-cAMP was plotted against the distance from the cut. Data for Neurobiotin was fit with a single exponential function. **(D)** Tracer diffusion in the horizontal cell layer w/o MFA preincubation, fit with exponential functions (solid line: Neurobiotin; dashed line: MFA+Neurobiotin). **(E)** Space constant of Neurobiotin spread was significantly lower after MFA preincubation than under control conditions (without MFA: 99 ± 43 µm, N = 25 cuts from 4 retinas, 4 mice; with MFA preincubation: 66 ± 25 µm, N = 14 cuts from 3 retinas, 3 mice; Mann-Whitney U test, p = 0.009, **). **(F)** The number of Neurobiotin+ horizontal cells/mm^2^ was significantly lower after MFA preincubation than under control conditions (without MFA: 312 ± 113 cells/mm^2^; with MFA preincubation: 187 ± 63 cells/mm^2^; Mann-Whitney U test, p = 0.0018, **).

In the outer retina of the mouse, horizontal cells are of the axon-bearing B-type (38). They form an extensive, gap junction-coupled network that is permeable to Neurobiotin (14). Therefore, we used Neurobiotin as a positive control before testing for the permeability of other Biotin-conjugated substances. As expected, Neurobiotin (**Figure 1A**) spread from the cutting site into the retina. The relative intensity of Neurobiotin+ cells decreased with distance from the cutting site (**Figures 1A, B**). This decay could be fit with a single exponential function as expected for a diffusion process (see a representative example in **Figure 1C**, R^2^ = 0.95, yielding a space constant of 107.5 µm, **Figure 1E**). To test whether the observed tracer spread was indeed mediated by gap junctions, retinas were pre-incubated in the gap junction blocker MFA (39). A relatively low concentration of 50 µM was used to prevent retina damage, which is not enough to block all gap junctions but is expected to reduce tracer spread between horizontal cells (40). As expected, MFA preincubation decreased the spread of Neurobiotin through the horizontal cell network (**Figures 1B, D**) and led to significantly lower space constants (**Figure 1E**; Neurobiotin: 99 ± 43 µm, N = 25 cuts from 4 retinas, 4 mice; Neurobiotin + MFA: 65 ± 25 µm, N = 14 cuts from 3 retinas, 3 mice; Mann-Whitney U test, p = 0.009). Also, the number of Neurobiotin+ cells/mm^2^ was significantly lower after MFA preincubation compared to control conditions (**Figure 1F**; Neurobiotin: 312 ± 113 cells/mm^2^; Neurobiotin + MFA: 187 ± 63 cells/mm^2^; Mann-Whitney U test, p = 0.0018).

**Figure 2:**
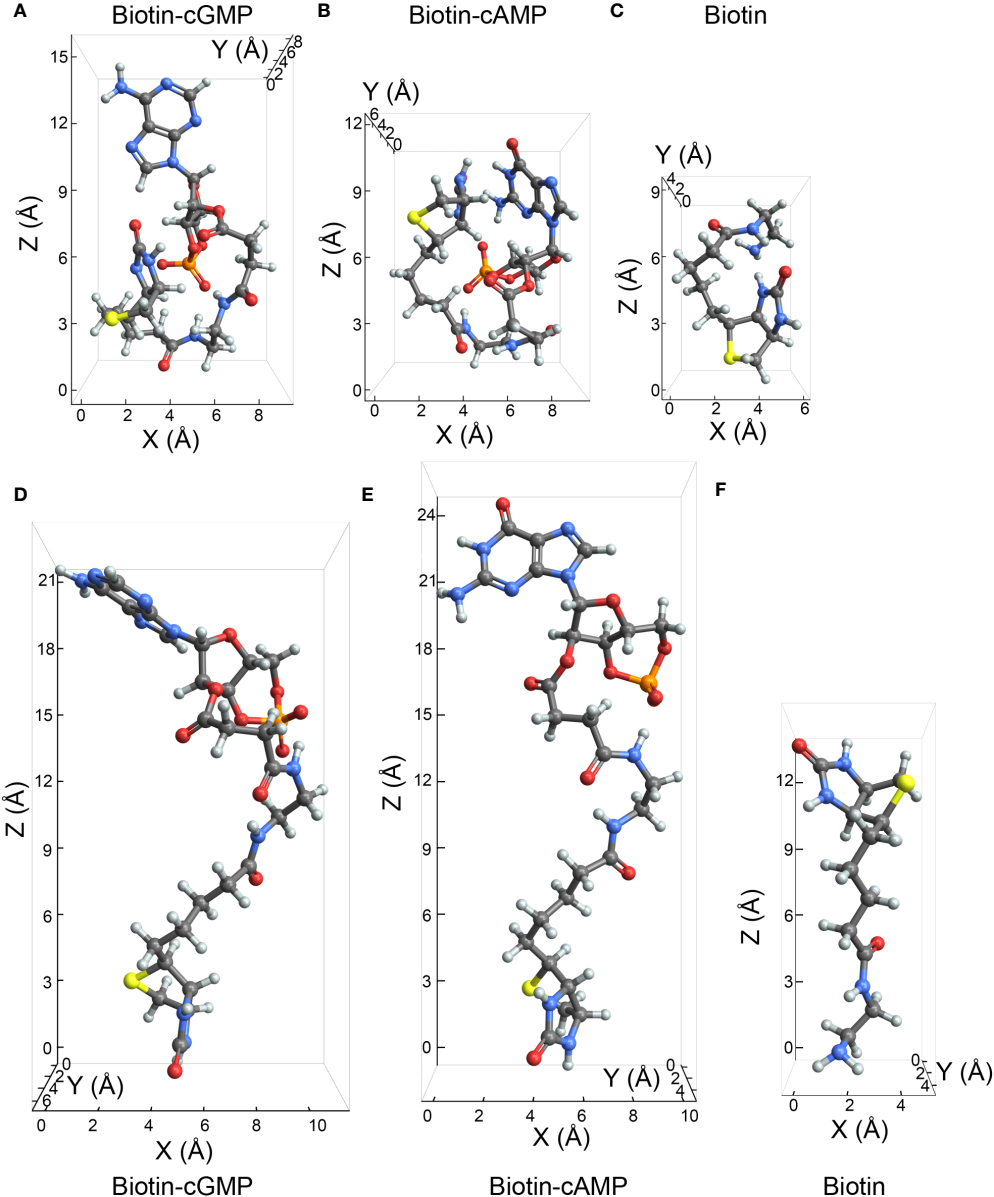
Characteristic sizes of Biotin-cGMP, Biotin-cAMP, and Biotin, followed by quantum chemical computations. The visualization presents the comparative sizes of Biotin-cGMP **(A, D)**, Biotin-cAMP **(B, E)**, and Biotin **(C, F)** in Cartesian coordinates. The Z-axis corresponds to the molecule’s longest dimension in these structures, while the X-axis represents the second-longest dimension, which is presumably the most relevant for the compounds’ permeability through gap junction channels. Structures **(A–C)**, derived from CREST analysis with dispersion correction, show that Biotin-cGMP has a maximum length (Z-axis) of 15.4 Å and a breadth (X-axis) of 9.1 Å. Similarly, Biotin-cAMP has a length of 11.9 Å and breadth of 9.3 Å, while Biotin measures 9.2 Å in length and 5.9 Å in breadth. Conversely, structures **(D–F)**, optimized without dispersion correction to qualitatively investigate the structure of Biotin-cAMP/-cGMP and Biotin geometries in solution, exhibit more elongated geometries. In these structures, Biotin-cGMP, Biotin-cAMP, and Biotin have the most extended dimensions of 20.9 Å, 24.0 Å, and 13.5 Å, respectively, with their respective breadths being 11.0 Å, 10.1 Å, and 5.0 Å.

After the successful establishment of a cut-loading protocol, we tested two Biotin-conjugated compounds for their permeability: Biotin-conjugated cGMP (MW = 832 g/mol) and Biotin-conjugated cAMP (MW = 826 g/mol). We determined the molecular dimensions of both substances (**Figures 2A, B, D, E**; **Supplementary Movie 1**, **2**) in comparison with Biotin (**Figures 2C, F**). To approximate the minimal pore diameter required to allow the substances’ permeation, we determined their second largest dimension (41) with dispersion correction (**Figures 2A–C**) and when inter-molecular dispersion interactions are possibly diminished by e.g., solvent (**Figures 2D–F**). The latter yielded breadths of 11.0 Å and 10.1 Å, respectively, for Biotin-cGMP and Biotin-cAMP which are considerably larger than the breadth of Biotin (5 Å).

After cut-loading, the two compounds did not spread through the horizontal cell network (**Figure 1A**), demonstrated by a very low number of Biotin+ cells distal from the cut. Accordingly, the relative intensity of the tracer spread could not be fit with an exponential function (**Figure 1C**). As hardly any Biotin+ horizontal cells were visible distal from the cut, we did not calculate the density of Biotin+ cells. Together, these results indicate that the gap junctions between horizontal cell dendrites are impermeable for the large compounds Biotin-cGMP and Biotin-cAMP.

### 3.2 Tracer coupling in the inner retina

**Figure 3:**
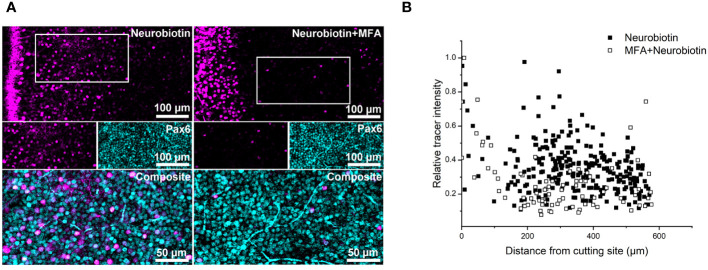
Various gap junctional networks in the amacrine cell layer contribute to the tracer decay. **(A)** Neurobiotin diffusion without (left) and with (right) MFA preincubation in the inner retina, cutting site on the left. Retinas were labeled with Pax6. Many Neurobiotin+ were Pax6+, confirming coupled cells as amacrine cells. White boxes indicate the areas shown as single channels in the middle panel; the overlay of both channels is shown as magnification in the bottom panel. **(B)** Relative tracer intensity of NB with and without MFA preincubation plotted against distance from cut. The large dispersal suggests that several gap junctional networks are present as the Neurobiotin spread does not follow an exponential function.

**Figure 4:**
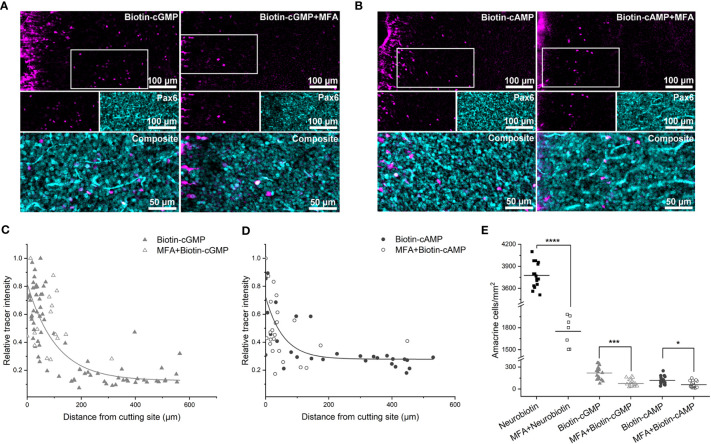
Gap junctions of some amacrine cells in the inner retina are permeable to Biotin-cGMP and -cAMP. **(A, B)** Biotin-cGMP **(A)** and Biotin-cAMP **(B)** diffusion in the inner retina without (left) and with (right) MFA preincubation. Pax6 labeling was used to identify coupled amacrine cells. White boxes indicate the areas shown as single channels in the middle panel; the overlay of both channels is shown as magnification in the bottom panel. **(C, D)** Diagrams show the relative tracer intensity of Pax6+ cells plotted against the distance from cut for Biotin-cGMP **(C)** and Biotin-cAMP **(D)**. Tracer diffusion followed an exponential function only under control conditions but not after MFA preincubation. **(E)** Summary diagram showing the number of Biotin+ and Pax6+ amacrine cells per mm^2^ for the different substances with and without MFA preincubation. Statistical analysis showed significant differences between Neurobiotin cuts with (n = 7 cuts from 2 retinas; 1752 ± 204 cells/mm^2^) and without MFA (N = 16 cuts from 3 retinas, 3 mice; 3777 ± 169 cells/mm^2^; p < 0.0001, ****), Biotin-cGMP cuts with (N = 24 from 3 retinas, 3 mice; 75 ± 44 cells/mm^2^) and without MFA (N = 16 cuts from 3 retinas, 3 mice; 219 ± 90 cells/mm^2^; p = 0.0001, ***), and Biotin-cAMP cuts with (N = 24 cuts from 3 retinas, 3 mice; 61 ± 39 cells/mm^2^) and without MFA (N = 16 cuts from 3 retinas, 3 mice; 118 ± 56 cells/mm^2^; p = 0.0255, *).

Next, we focused on the inner retina where many different gap junction-coupled networks exist, e.g., narrow-field AII amacrine cells coupled among each other and to ON bipolar cells (42), various types of wide-field amacrine cells coupled among each other (43, 44) and to ganglion cells (45). Again, we first analyzed samples cut-loaded with Neurobiotin (**Figure 3A**). To restrict our analysis to amacrine cells, we co-labeled the retinas for Pax6, a marker for amacrine but not bipolar and Müller cells in the mouse retina (46). Many Neurobiotin+/Pax6+ cells became visible. However, tracer intensity did not decay exponentially with distance from the cutting site (**Figure 3B**). We hypothesize that the many gap junctional networks with various gap junction proteins and cell types connected obscure the exponential decay. To test for gap junctional coupling, we preincubated retinas with the gap junction blocker MFA (**Figures 3**, **4**). As we could not determine length constants, we counted the Neurobiotin+/Pax6+ cells. MFA was reported to block gap junctions between AII amacrine cells (47), representing the most numerous amacrine cell types in the mouse retina (48, 49). Even though MFA will not block all AII gap junctions at a concentration of 50 µM (47), MFA preincubation should substantially reduce Neurobiotin+/Pax6+ amacrine cells. This was indeed the case. The number of Neurobiotin+/Pax6+ cells significantly decreased from 3777 ± 169 cells/mm^2^ (N = 16 cuts from 3 retinas, 3 mice) in control to 1752 ± 204 cells/mm^2^ in MFA-treated retinas (N = 7 cuts from 2 retinas, 2 mice, **Figure 4E**, one-way ANOVA, p < 0.0001).

Next, we tested the two Biotin-conjugated compounds, Biotin-cGMP and -cAMP, and again focused our analysis on Biotin+/Pax6+ cells (**Figure 4**). Both substances showed weak coupling (**Figures 4A, B**), with 219 ± 90 cells per mm^2^ for Biotin-cGMP (N = 16 cuts from 3 retinas, 3 mice) and 118 ± 56 cells per mm^2^ for Biotin-cAMP (N = 16 cuts from 3 retinas, 3 mice). As the coupling decayed exponentially from the cutting site (**Figures 4C, D**), Biotin-cGMP and -cAMP may only pass through the gap junctions of a single cell type. Differences between the two compounds were significant (**Figure 4E**, one-way ANOVA, p = 0.0111). Coupling was significantly lower than Neurobiotin (one-way ANOVA, p < 0.0001 for Biotin-cGMP and Neurobiotin, p < 0.0001 for Biotin-cAMP and Neurobiotin). It significantly decreased for both Biotin-conjugated compounds after MFA preincubation (**Figure 4E**, one-way ANOVA, p = 0.0001 for Biotin-cGMP and p = 0.0255 for Biotin-cAMP), confirming the involvement of gap junction-mediated substance transfer. MFA preincubation seemed to reduce substance transfer to a background level, as differences between Biotin-cGMP and -cAMP after MFA treatment were insignificant (one-way ANOVA, p = 0.9765).

**Figure 5:**
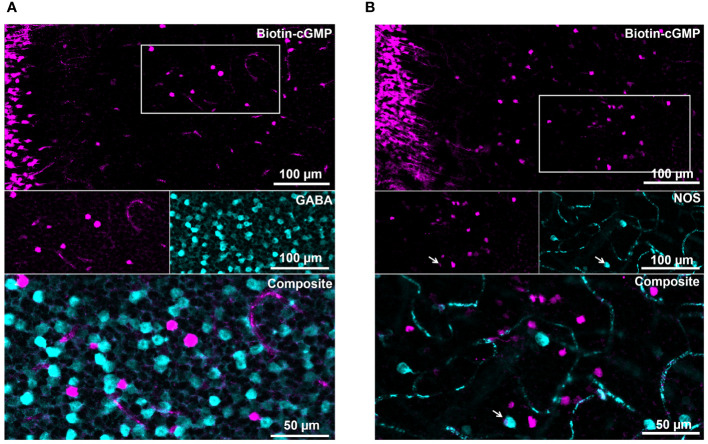
The amacrine cell type permeable for the large Biotin-conjugated compounds are GABA- and NOS-. **(A, B)** Retinas cut-loaded with Biotin-cGMP were co-stained with GABA **(A)** and NOS1 **(B)**, a marker for nitric oxide-synthesizing amacrine cells. White boxes indicate the areas shown as single channels in the middle panel; the overlay of both channels is shown as magnification in the bottom panel. Biotin+ cells did not colocalize with GABA or NOS, suggesting that coupled cells belong to non-GABAergic amacrine cells despite their relatively low density. The arrow points to a seemingly colocalized cell but closer inspection of the confocal stack showed that the NOS+ cell lies on top of the Biotin+ cell.

Together, these results suggest that the large compounds Biotin-cGMP and -cAMP can pass the gap junctions of at least one class of amacrine cells in the inner retina. As the number of coupled cells/mm^2^ is relatively low, this cell class does not comprise narrow-field AII amacrine cells but likely represents a class of wide-field amacrine cells. To test this hypothesis, we co-labeled coupled cells with GABA (**Figure 5A**). We found that amacrine cells with gap junctions permeable for Biotin conjugates are all GABA- and may represent narrow-field amacrine cells. In addition, we labeled for NO synthase (NOS) because NOS-expressing amacrine cells were reported to form a strongly coupled gap junctional network and have a similar density (50) as the Biotin+ cells we found. However, Biotin+ cells were all NOS- and thus do not seem to represent this cell type (**Figure 5B**).

## 4 Discussion

This study used the mouse retina to study whether large Biotin-conjugated substances with a molecular mass of >0.8 kDa can pass neuronal gap junctions. Indeed, we found that a network of amacrine cells in the inner retina expresses gap junctions, which allow the passage of these compounds, suggesting that neuronal gap junctions may be exploited to transfer of large substances with therapeutic value (e.g., miRNAs).

### 4.1 Gap junctions of horizontal cells are impermeable to Biotin-cGMP and -cAMP

We successfully adopted a cut-loading method (40, 51) and validated it with Neurobiotin, a tracer known to permeate through retinal gap junctions (4), and MFA, a well-characterized blocker of gap junctions (39). As expected, Neurobiotin spread through gap junctions in horizontal cells (14, 52) and accumulated in horizontal cell somata without clearly visualizing the dendro-dendritic network. In line with expectation, Neurobiotin spread was strongly decreased when the retina was preincubated with MFA (39), demonstrating the functioning of the assay. However, both Biotin-conjugated cyclic nucleotides could not pass the gap junctional network of horizontal cell dendrites. This aligns with earlier work showing that Lucifer Yellow (0.4424 kDa) cannot pass gap junctions made of connexin57 (53), which is the connexin forming the dendro-dendritic gap junctions in mouse horizontal cells (14, 54, 55). Mouse horizontal cells were reported to express a second connexin, connexin50 (56), which has a much higher single-channel conductance than connexin57 (220 pS *vs*. 57 pS, respectively) (57, 58) and can pass Lucifer Yellow as shown in A-type horizontal cells of the rabbit retina (59). However, connexin50 was only detected in axo-axonal gap junctions of the B-type horizontal cells in the mouse retina (56), which were not probed by the cut-loading method as we never saw any labeled axon terminals of horizontal cells in our cut-loading experiments.

### 4.2 Some amacrine cells form gap junctions large enough for Biotin-cGMP and -cAMP passage

In the inner retina, the high density of Neurobiotin+ cells suggested that the cut-loading method probed many different gap junction networks simultaneously. Gap junctional coupling between amacrine cells and bipolar cells [predominantly by AII amacrine cells (60)], between amacrine cells [again, predominantly by AII amacrine cells (60)], and between amacrine and ganglion cells (45) may obscure exponential decay of tracer intensity from the cutting edge. Therefore, we could not determine length constants to estimate diffusion, so we counted the number of cells per mm^2^ to identify the cells (and potentially the gap junction protein or connexin) underlying the coupling. In controls, more than 3000 cells/m^2^ were Neurobiotin+, in line with the assumption that most Neurobiotin+ cells represent AII amacrine cells because they have a density between 3000 and 3800 cells/m^2^. AII cells form gap junctions made of connexin36 (7), which likely did not allow the Biotin-conjugated cyclic nucleotides to pass because the number of coupled cells dropped dramatically when using these tracers. This is not surprising as connexin36 channels have a very low unitary conductance [10-15 pS (61)]. However, unitary conductance might not always be informative on the permeability of gap junctions because, despite their low unitary conductance, connexin36 gap junctions can pass Lucifer Yellow (61) whereas, for example, connexin57 with its much higher unitary conductance cannot (53).

Biotin-cGMP and -cAMP showed some gap junction coupling in amacrine cells, as evidenced by 1) co-labeling of Biotin+ with Pax6 in the proximal inner nuclear layer and 2) decrease in the number of Biotin+/Pax6+ cells after MFA preincubation although we cannot fully exclude the possibility that tracer uptake by wide-field amacrine cells weakly contributes to the number of Biotin+ cells. In a retina, which was cut in only one position with a Biotin-cAMP-coated blade, Biotin+ cells extended well beyond the presumed size of individual wide-field amacrine cells and were detected also up to 1 mm from the cutting site (**Supplementary Figure 2**). Based on the relatively low density of Biotin+/Pax6+ cells (≤ 200 cells/m^2^), we suspected these cells to represent wide-field amacrine cells, which are usually GABAergic (62). Yet, double-labeling with GABA revealed that all coupled cells were GABA-. We also labeled for NO synthase because an earlier study showed that amacrine cells expressing NO synthase (called nNOS-2 amacrine cells) form large coupled networks, made of connexin45 (50). However, all Biotin+ amacrine cells were NOS1-, also excluding this cell type. As the density of Biotin+/Pax6+ cells seems too low for glycinergic amacrine cells, which are narrow-field amacrine cells (62, 63), these cells may represent GABA-/glycine- amacrine cells (also termed nGnG). Recently, four types of nGnG cells have been identified in a transcriptomic approach and reported to have a low cell density (46). Interestingly, one of the nGnG amacrine cells shows extensive tracer coupling and expresses various connexins: connexin36, connexin45, and connexin23 (64). It seems unlikely that connexin36 and connexin45 let Biotin-conjugated cyclic nucleotides pass in one cell type and not in another, but we cannot completely exclude this possibility. However, connexin23 may be an exciting candidate; it was reported not to form functional gap junction channels *in vitro* but hemichannels permeable to ATP (65), which is rather large (0.51 kDa). Yet, its involvement in tracer coupling has never been reported, and protein expression was never shown for the retina.

In summary, some amacrine cells are able to transfer large Biotin-conjugated substances via gap junctions from one cell to another but which of the more than 60 different types of amacrine cells (46) is responsible for this, remains elusive.

There seemed to be a slight preference for Biotin-cGMP passage over Biotin-cAMP despite the relatively small difference in molecular weight (0.006 kDa). In fact, our calculations of the second largest dimension showed that Biotin-cGMP is slightly larger than Biotin-cAMP with dispersion correction (**Figures 2A, B**) and when inter-molecular dispersion interactions are limited by e.g., solvent (**Figures 2D, E**) while both substances are considerably larger than Biotin (**Figures 2C, F**). Although the Biotin-conjugated cyclic nucleotides were very long (>20 Å), their second largest dimension was similar to other gap junction-permeable substances, like Lucifer Yellow [9.9 Å (66)] and Alexa 488 [10.5 Å (41)] and even smaller than Alexa 594 [13.8 Å (41)].

### 4.3 Potential exploitation of the results

In recent years, gap-junction-mediated coupling was not only found to facilitate or counteract cell death processes but also demonstrated as a tool to deliver substances of potential therapeutic value (miRNAs) in cultured cells expressing connexin43 (25, 27). These miRNAs have a size of ~1 kDa. Our results on the transfer of rather large compounds in the mouse retina may suggest that even in intact neuronal tissue, some gap junctions may be permeable to miRNAs or siRNAs. This would open up exciting possibilities for neuron protection: Amacrine cells undergo apoptosis after retinal ganglion cells in ischemic retinas due to a gap-junction-mediated bystander effect (16). Delivering, for example, siRNAs interfering with apoptotic pathways may offer the potential to prevent progressive cell loss in retinal degenerative diseases. However, the size of a compound is not the only determinant for its ability to permeate through gap junctions, but gap junction composition, phosphorylation state, transjunctional voltage, pore size, and electrostatic properties come into play (1, 41, 66–68). Therefore, further studies are needed to explore the therapeutic potential of health signal delivery through retinal gap junctions.

## Data availability statement

The raw data supporting the conclusions of this article will be made available by the authors, without undue reservation.

## Ethics statement

The animal study was approved by Niedersächsisches Landesamt für Verbraucherschutz und Lebensmittelsicherheit. The study was conducted in accordance with the local legislation and institutional requirements.

## Author contributions

CY: Data curation, Formal analysis, Visualization, Writing – review & editing. LG: Data curation, Formal analysis, Visualization, Writing – review & editing. IS: Data curation, Formal analysis, Funding acquisition, Visualization, Writing – review & editing. KD: Conceptualization, Funding acquisition, Project administration, Resources, Supervision, Writing – original draft.

## References

[1] Loewenstein WR. Junctional intercellular communication and the control of growth. Biochim Biophys Acta (BBA) - Rev Cancer (1979) 560:1–65. doi: 10.1016/0304-419X(79)90002-7 216404

[2] Furshpan EJ Potter DD. Low-resistance junctions between cells in embryos and tissue culture. Curr Top Dev Biol (1968) 3:95–127. doi: 10.1016/s0070-2153(08)60352-x 4331697

[3] Bloomfield SA Völgyi B. The diverse functional roles and regulation of neuronal gap junctions in the retina. Nat Rev Neurosci (2009) 10:495–506. doi: 10.1038/nrn2636 19491906 PMC3381350

[4] Vaney DI. Many diverse types of retinal neurons show tracer coupling when injected with biocytin or Neurobiotin. Neurosci Lett (1991) 125:187–90. doi: 10.1016/0304-3940(91)90024-n 1715532

[5] Naus CC Bechberger JF Caveney S Wilson JX. Expression of gap junction genes in astrocytes and C6 glioma cells. Neurosci Lett (1991) 126:33–6. doi: 10.1016/0304-3940(91)90364-y 1650934

[6] Zahs KR Kofuji P Meier C Dermietzel R. Connexin immunoreactivity in glial cells of the rat retina. J Comp Neurol (2003) 455:531–46. doi: 10.1002/cne.10524 12508325

[7] Güldenagel M Ammermüller J Feigenspan A Teubner B Degen J Söhl G. Visual transmission deficits in mice with targeted disruption of the gap junction gene connexin36. J Neurosci (2001) 21:6036–44. doi: 10.1523/JNEUROSCI.21-16-06036.2001 PMC6763178 11487627

[8] Deans MR Volgyi B Goodenough DA Bloomfield SA Paul DL. Connexin36 is essential for transmission of rod-mediated visual signals in the mammalian retina. Neuron (2002) 36:703–12. doi: 10.1016/S0896-6273(02)01046-2 PMC2834592 12441058

[9] Maxeiner S Dedek K Janssen-Bienhold U Ammermüller J Brune H Kirsch T. Deletion of connexin45 in mouse retinal neurons disrupts the rod/cone signaling pathway between AII amacrine and ON cone bipolar cells and leads to impaired visual transmission. J Neurosci (2005) 25:566–76. doi: 10.1523/JNEUROSCI.3232-04.2005 PMC6725315 15659592

[10] Hornstein EP Verweij J Li PH Schnapf JL. Gap-junctional coupling and absolute sensitivity of photoreceptors in macaque retina. J Neurosci (2005) 25:11201–9. doi: 10.1523/JNEUROSCI.3416-05.2005 PMC6725652 16319320

[11] DeVries SH Qi X Smith R Makous W Sterling P. Electrical coupling between mammalian cones. Curr Biol (2002) 12:1900–7. doi: 10.1016/S0960-9822(02)01261-7 12445382

[12] Völgyi B Pan F Paul DL Wang JT Huberman AD Bloomfield SA. Gap junctions are essential for generating the correlated spike activity of neighboring retinal ganglion cells. PloS One (2013) 8:e69426. doi: 10.1371/journal.pone.0069426 23936012 PMC3720567

[13] Brivanlou IH Warland DK Meister M. Mechanisms of concerted firing among retinal ganglion cells. Neuron (1998) 20:527–39. doi: 10.1016/s0896-6273(00)80992-7 9539126

[14] Shelley J Dedek K Schubert T Feigenspan A Schultz K Hombach S. Horizontal cell receptive fields are reduced in connexin57-deficient mice. Eur J Neurosci (2006) 23:3176–86. doi: 10.1111/j.1460-9568.2006.04848.x 16820008

[15] Muller JF Dacheux RF. Alpha ganglion cells of the rabbit retina lose antagonistic surround responses under dark adaptation. Vis Neurosci (1997) 14:395–401. doi: 10.1017/s0952523800011512 9147490

[16] Akopian A Atlasz T Pan F Wong S Zhang Y Völgyi B. Gap junction-mediated death of retinal neurons is connexin and insult specific: a potential target for neuroprotection. J Neurosci (2014) 34:10582–91. doi: 10.1523/JNEUROSCI.1912-14.2014 PMC4200109 25100592

[17] Paschon V Higa GSV Resende RR Britto LRG Kihara AH. Blocking of connexin-mediated communication promotes neuroprotection during acute degeneration induced by mechanical trauma. PloS One (2012) 7:e45449. doi: 10.1371/journal.pone.0045449 23029016 PMC3447938

[18] Freeman SM Abboud CN Whartenby KA Packman CH Koeplin DS Moolten FL. The “bystander effect”: tumor regression when a fraction of the tumor mass is genetically modified. Cancer Res (1993) 53:5274–83. 8221662

[19] Naus CC Ozog MA Bechberger JF Nakase T. A neuroprotective role for gap junctions. Cell Commun Adhes (2001) 8:325–8. doi: 10.3109/15419060109080747 12064612

[20] Hutnik CML Pocrnich CE Liu H Laird DW Shao Q. The protective effect of functional connexin43 channels on a human epithelial cell line exposed to oxidative stress. Invest Ophthalmol Vis Sci (2008) 49:800–6. doi: 10.1167/iovs.07-0717 18235030

[21] Striedinger K Petrasch-Parwez E Zoidl G Napirei M Meier C Eysel UT. Loss of connexin36 increases retinal cell vulnerability to secondary cell loss. Eur J Neurosci (2005) 22:605–16. doi: 10.1111/j.1460-9568.2005.04228.x 16101742

[22] Laird DW Lampe PD. Therapeutic strategies targeting connexins. Nat Rev Drug Discovery (2018) 17:905–21. doi: 10.1038/nrd.2018.138 PMC6461534 30310236

[23] Gadok AK Busch DJ Ferrati S Li B Smyth HDC Stachowiak JC. Connectosomes for direct molecular delivery to the cellular cytoplasm. J Am Chem Soc (2016) 138:12833–40. doi: 10.1021/jacs.6b05191 27607109

[24] Gadok AK Zhao C Meriwether AI Ferrati S Rowley TG Zoldan J. Display of single-domain antibodies on the surfaces of connectosomes enables gap junction mediated drug delivery to specific cell populations. Biochemistry (2018) 57:81–90. doi: 10.1021/acs.biochem.7b00688 28829120 PMC5880529

[25] Brink PR Valiunas V Gordon C Rosen MR Cohen IS. Can gap junctions deliver? Biochim Biophys Acta (2012) 1818:2076–81. doi: 10.1016/j.bbamem.2011.09.025 21986484

[26] Trementozzi AN Hufnagel S Xu H Hanafy MS Rosero Castro F Smyth HDC. Gap junction liposomes for efficient delivery of chemotherapeutics to solid tumors. ACS Biomater Sci Eng (2020) 6:4851–7. doi: 10.1021/acsbiomaterials.0c01047 PMC8483596 33455217

[27] Valiunas V Polosina YY Miller H Potapova IA Valiuniene L Doronin S. Connexin-specific cell-to-cell transfer of short interfering RNA by gap junctions. J Physiol (2005) 568:459–68. doi: 10.1113/jphysiol.2005.090985 PMC1474730 16037090

[28] Steinle JJ. Review: Role of cAMP signaling in diabetic retinopathy. Mol Vis (2020) 26:355–8. PMC7245604 32476815

[29] Sondereker KB Stabio ME Jamil JR Tarchick MJ Renna JM. Where you cut matters: A dissection and analysis guide for the spatial orientation of the mouse retina from ocular landmarks. J Vis Exp (2018) (138):57861. doi: 10.3791/57861 30124662 PMC6126625

[30] Schindelin J Arganda-Carreras I Frise E Kaynig V Longair M Pietzsch T. Fiji: an open-source platform for biological-image analysis. Nat Methods (2012) 9:676–82. doi: 10.1038/nmeth.2019 PMC3855844 22743772

[31] Neese F. Software update: The ORCA program system—Version 5.0. WIREs Comput Mol Sci (2022) 12:e1606. doi: 10.1002/wcms.1606

[32] Weigend F Ahlrichs R. Balanced basis sets of split valence, triple zeta valence and quadruple zeta valence quality for H to Rn: Design and assessment of accuracy. Phys Chem Chem Phys (2005) 7:3297–305. doi: 10.1039/B508541A 16240044

[33] Rappoport D Furche F. Property-optimized Gaussian basis sets for molecular response calculations. J Chem Phys (2010) 133:134105. doi: 10.1063/1.3484283 20942521

[34] Grimme S Antony J Ehrlich S Krieg H. A consistent and accurate ab initio parametrization of density functional dispersion correction (DFT-D) for the 94 elements H-Pu. J Chem Phys (2010) 132:154104. doi: 10.1063/1.3382344 20423165

[35] Grimme S Ehrlich S Goerigk L. Effect of the damping function in dispersion corrected density functional theory. J Comput Chem (2011) 32:1456–65. doi: 10.1002/jcc.21759 21370243

[36] Pracht P Bohle F Grimme S. Automated exploration of the low-energy chemical space with fast quantum chemical methods. Phys Chem Chem Phys (2020) 22:7169–92. doi: 10.1039/C9CP06869D 32073075

[37] Grimme S. Exploration of chemical compound, conformer, and reaction space with meta-dynamics simulations based on tight-binding quantum chemical calculations. J Chem Theory Comput (2019) 15:2847–62. doi: 10.1021/acs.jctc.9b00143 30943025

[38] Peichl L González-Soriano J. Morphological types of horizontal cell in rodent retinae: a comparison of rat, mouse, gerbil, and Guinea pig. Vis Neurosci (1994) 11:501–17. doi: 10.1017/S095252380000242X 8038125

[39] Pan F Mills SL Massey SC. Screening of gap junction antagonists on dye coupling in the rabbit retina. Vis Neurosci (2007) 24:609–18. doi: 10.1017/S0952523807070472 PMC2213422 17711600

[40] Myles WE McFadden SA. Analytical methods for assessing retinal cell coupling using cut-loading. PloS One (2022) 17:e0271744. doi: 10.1371/journal.pone.0271744 35853039 PMC9295955

[41] Weber PA Chang H-C Spaeth KE Nitsche JM Nicholson BJ. The permeability of gap junction channels to probes of different size is dependent on connexin composition and permeant-pore affinities. Biophys J (2004) 87:958–73. doi: 10.1529/biophysj.103.036350 PMC1304503 15298902

[42] Strettoi E Raviola E Dacheux RF. Synaptic connections of the narrow-field, bistratified rod amacrine cell (AII) in the rabbit retina. J Comp Neurol (1992) 325:152–68. doi: 10.1002/cne.903250203 1460111

[43] Brüggen B Meyer A Boven F Weiler R Dedek K. Type 2 wide-field amacrine cells in TH::GFP mice show a homogenous synapse distribution and contact small ganglion cells. Eur J Neurosci (2014) 41(6):734–47. doi: 10.1111/ejn.12813 25546402

[44] Knop GC Pottek M Monyer H Weiler R Dedek K. Morphological and physiological properties of enhanced green fluorescent protein (EGFP)-expressing wide-field amacrine cells in the ChAT-EGFP mouse line. Eur J Neurosci (2014) 39:800–10. doi: 10.1111/ejn.12443 24299612

[45] Völgyi B Chheda S Bloomfield SA. Tracer coupling patterns of the ganglion cell subtypes in the mouse retina. J Comp Neurol (2009) 512:664–87. doi: 10.1002/cne.21912 PMC3373319 19051243

[46] Yan W Laboulaye MA Tran NM Whitney IE Benhar I Sanes JR. Mouse retinal cell atlas: molecular identification of over sixty amacrine cell types. J Neurosci (2020) 40:5177–95. doi: 10.1523/JNEUROSCI.0471-20.2020 PMC7329304 32457074

[47] Veruki ML Hartveit E. Meclofenamic acid blocks electrical synapses of retinal AII amacrine and ON-cone bipolar cells. J Neurophysiol (2008) 101:2339–47. doi: 10.1152/jn.00112.2009 19279153

[48] Rice D Curran T. Disabled-1 is expressed in type AII amacrine cells in the mouse retina. J Comp Neurol (2000) 424:327–38. doi: 10.1002/1096-9861(20000821)424:2<327::AID-CNE10>3.0.CO;2-6 10906706

[49] Meyer A Tetenborg S Greb H Segelken J Dorgau B Weiler R. Connexin30.2: *in vitro* interaction with connexin36 in heLa cells and expression in AII amacrine cells and intrinsically photosensitive ganglion cells in the mouse retina. Front Mol Neurosci (2016) 9:36. doi: 10.3389/fnmol.2016.00036 27303262 PMC4882342

[50] Jacoby J Nath A Jessen ZF Schwartz GW. A self-regulating gap junction network of amacrine cells controls nitric oxide release in the retina. Neuron (2018) 100:1149–1162.e5. doi: 10.1016/j.neuron.2018.09.047 30482690 PMC6317889

[51] Choi HJ Ribelayga CP Mangel SC. Cut-loading: a useful tool for examining the extent of gap junction tracer coupling between retinal neurons. J Vis Exp (2012) (59):3180. doi: 10.3791/3180 22269968 PMC3462560

[52] Trümpler J Dedek K Schubert T de Sevilla Müller LP Seeliger M Humphries P. Rod and cone contributions to horizontal cell light responses in the mouse retina. J Neurosci (2008) 28:6818–25. doi: 10.1523/JNEUROSCI.1564-08.2008 PMC6670969 18596157

[53] Manthey D Bukauskas F Lee CG Kozak CA Willecke K. Molecular cloning and functional expression of the mouse gap junction gene connexin-57 in human HeLa cells. J Biol Chem (1999) 274:14716–23. doi: 10.1074/jbc.274.21.14716 10329667

[54] Hombach S Janssen-Bienhold U Söhl G Schubert T Büssow H Weiler R. Functional expression of connexin57 in horizontal cells of the mouse retina. Eur J Neurosci (2004) 19:2633–40. doi: 10.1111/j.0953-816X.2004.03360.x 15147297

[55] Janssen-Bienhold U Trümpler J Hilgen G Schultz K Pérez de Sevilla Müller L Sonntag S. Connexin57 is expressed in dendro-dendritic and axo-axonal gap junctions of mouse horizontal cells and its distribution is modulated by light. J Comp Neurol (2009) 513:363–74. doi: 10.1002/cne.21965 19177557

[56] Dorgau B Herrling R Schultz K Greb H Segelken J Ströh S. Connexin50 couples axon terminals of mouse horizontal cells by homotypic gap junctions. J Comp Neurol (2015) 523:2062–81. doi: 10.1002/cne.23779 25823610

[57] Hopperstad MG Srinivas M Spray DC. Properties of gap junction channels formed by Cx46 alone and in combination with Cx50. Biophys J (2000) 79:1954–66. doi: 10.1016/S0006-3495(00)76444-7 PMC1301086 11023900

[58] Palacios-Prado N Sonntag S Skeberdis VA Willecke K Bukauskas FF. Gating, permselectivity and pH-dependent modulation of channels formed by connexin57, a major connexin of horizontal cells in the mouse retina. J Physiol (Lond) (2009) 587:3251–69. doi: 10.1113/jphysiol.2009.171496 PMC2727035 19433576

[59] O’Brien JJ Li W Pan F Keung J O’Brien J Massey SC. Coupling between A-type horizontal cells is mediated by connexin 50 gap junctions in the rabbit retina. J Neurosci (2006) 26:11624–36. doi: 10.1523/JNEUROSCI.2296-06.2006 PMC6674794 17093084

[60] Hartveit E Veruki ML. Electrical synapses between AII amacrine cells in the retina: Function and modulation. Brain Res (2012) 1487:160–72. doi: 10.1016/j.brainres.2012.05.060 22776293

[61] Srinivas M Rozental R Kojima T Dermietzel R Mehler M Condorelli DF. Functional properties of channels formed by the neuronal gap junction protein connexin36. J Neurosci (1999) 19:9848–55. doi: 10.1523/JNEUROSCI.19-22-09848.1999 PMC6782942 10559394

[62] Werblin FS. The retinal hypercircuit: a repeating synaptic interactive motif underlying visual function. J Physiol (Lond) (2011) 589:3691–702. doi: 10.1113/jphysiol.2011.210617 PMC3171878 21669978

[63] Wässle H Heinze L Ivanova E Majumdar S Weiss J Harvey RJ. Glycinergic transmission in the mammalian retina. Glra1 (2009) 2:6.2009. doi: 10.3389/neuro.02.006.2009 PMC2777502 19924257

[64] Kerstein PC Leffler J Sivyer B Taylor WR Wright KM. Gbx2 identifies two amacrine cell subtypes with distinct molecular, morphological, and physiological properties. Cell Rep (2020) 33:108382. doi: 10.1016/j.celrep.2020.108382 33207201 PMC7713908

[65] Sonntag S Söhl G Dobrowolski R Zhang J Theis M Winterhager E. Mouse lens connexin23 (Gje1) does not form functional gap junction channels but causes enhanced ATP release from HeLa cells. Eur J Cell Biol (2009) 88:65–77. doi: 10.1016/j.ejcb.2008.08.004 18849090 PMC2719720

[66] Kanaporis G Brink PR Valiunas V. Gap junction permeability: selectivity for anionic and cationic probes. Am J Physiology-Cell Physiol (2011) 300:C600–9. doi: 10.1152/ajpcell.00316.2010 PMC3063972 21148413

[67] Harris AL. Connexin channel permeability to cytoplasmic molecules. Prog Biophysics Mol Biol (2007) 94:120–43. doi: 10.1016/j.pbiomolbio.2007.03.011 PMC1995164 17470375

[68] Goldberg GS Valiunas V Brink PR. Selective permeability of gap junction channels. Biochim Biophys Acta (BBA) - Biomembranes (2004) 1662:96–101. doi: 10.1016/j.bbamem.2003.11.022 15033581

