# Supplementary material for: Biotin-cGMP and -cAMP are able to permeate through the gap junctions of some amacrine cells in the mouse retina despite their large size
Source: Front Ophthalmol (Lausanne). 2024 Jan 15;3:1334602. doi: 10.3389/fopht.2023.1334602 (PMC11182161; doi:10.3389/fopht.2023.1334602)
Supplement: Supplementary file 1 [file DataSheet_1.pdf]

Supplementary Material

Biotin-cGMP and -cAMP are able to permeate through the gap junctions of some amacrine cells in the mouse retina despite their large size

Chunxu Yuan[1], Luca Gerhards[2], Ilia A. Solov'yov[2,3,4], Karin Dedek[1,3*]

[1]Animal Navigation, Institute for Biology and Environmental Sciences, Carl von Ossietzky Universität Oldenburg, 26111 Oldenburg, Germany

[2]Institute of Physics, Carl von Ossietzky Universität Oldenburg, 26111 Oldenburg, Germany

[3]Research Center Neurosensory Science, University of Oldenburg, Oldenburg, Germany

[4]CeNaD – Center for Nanoscale Dynamics, University of Oldenburg, 26111 Oldenburg, Germany

*** Correspondence:**
Karin Dedek
karin.dedek@uol.de

1 Supplementary Data

Supplementary Video 1: Animation of Biotin-cGMP and Biotin-cAMP, and Biotin. The visualization presents the comparative sizes of Biotin-cGMP and Biotin-cAMP. Structures, derived from CREST analysis with dispersion correction, exhibit more stacked geometries compared to geometries without correction (Supplementary Video 2).

Supplementary Video 2: Animation of Biotin-cGMP and Biotin-cAMP. The visualization presents the comparative sizes of Biotin-cGMP and Biotin-cAMP. Structures, optimized without dispersion correction to qualitatively investigate the structure of Biotin-cAMP/-cGMP and Biotin geometries when inter-molecular dispersion interactions are possibly diminished by e.g., solvent. As can be observed the structures are more elongated compared to the dispersion corrected case (Supplementary Video 1).

2 Supplementary Figures and Tables

2.1 Supplementary Figures

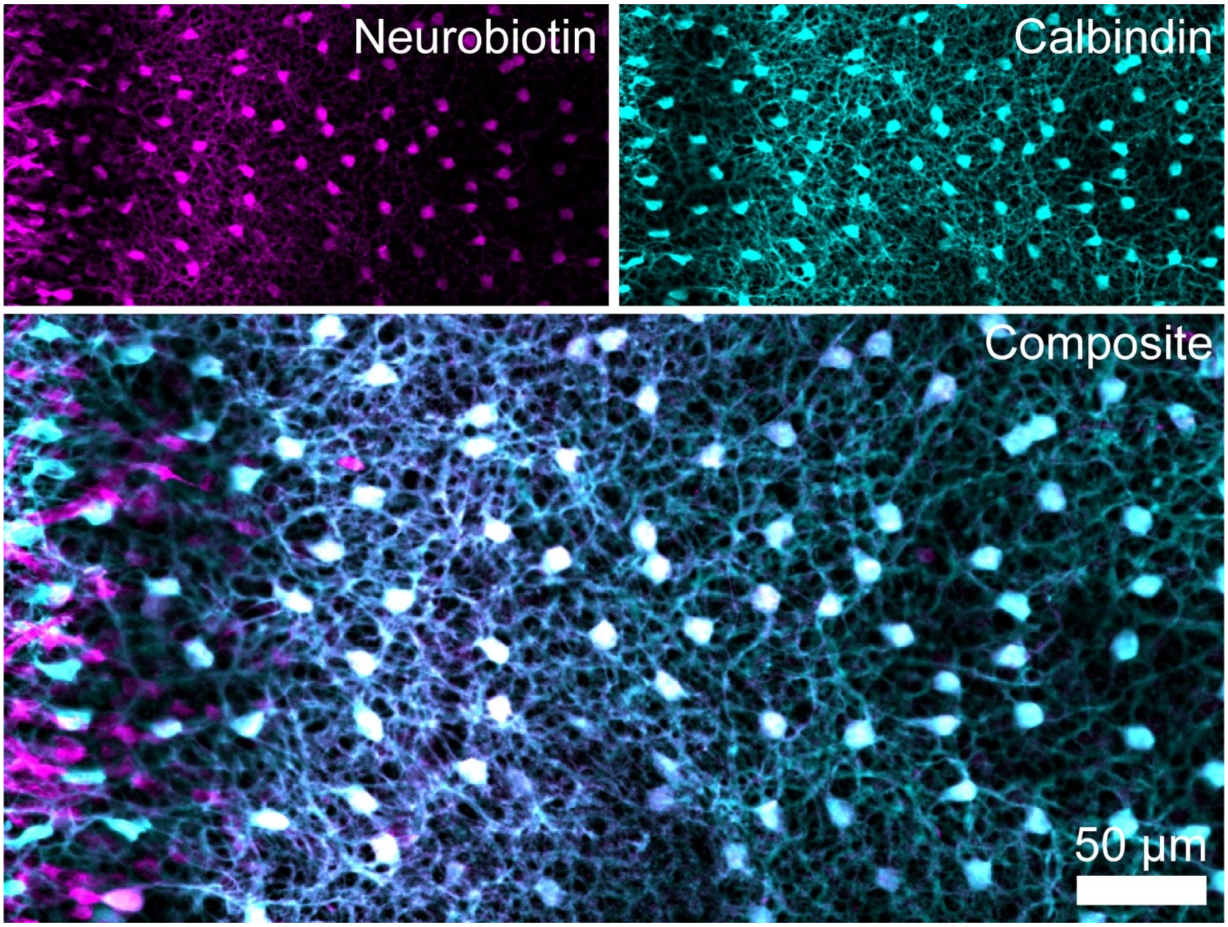

Supplementary Figure 1: Identification of horizontal cells. Horizontal cells were identified based on their large somata, their regular spacing and soma position in the distal inner nuclear layer. Calbindin as a marker for mouse horizontal cells was used to confirm the cell identity in one experiment.

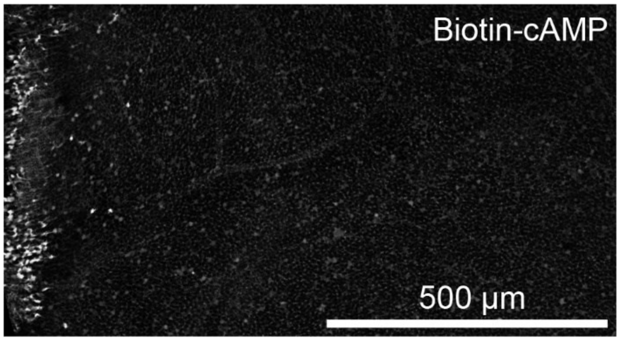

Supplementary Figure 2: Biotin-cAMP diffusion occurs over a large distance. Diffusion of Biotin-cAMP in the inner retina, cutting site on the left. Numerous cells are Biotin+ >500 µm away from the cutting site, suggesting gap junction-mediated spread of Biotin-cAMP rather than tracer uptake by cut dendrites of wide-field amacrine cells.
